# Positionspapier der Berlin-Brandenburgischen Chirurgischen Gesellschaft – zur Zukunft der chirurgischen Weiterbildung

**DOI:** 10.1007/s00104-024-02071-4

**Published:** 2024-04-24

**Authors:** Karl H. Hillebrandt, Eva Dobrindt, Felix Krenzien, Simon Moosburner, Johann Pratschke, Teilnehmer der Neuhardenberger Gespräche in Vertretung der Berlin-Brandenburgischen Chirurgischen Gesellschaft, Ulrich Adam, Georg Bauer, Christoph Benckert, Mike Bereuter, Katharina Beyer, Peter Bobbert, Joachim Böttger, Yao Chen, Lope Estévez Schwarz, Stefan Farke, Ullrich Fleck, Marek Frakowiak, Georg Fritzsch, Klaus Gellert, Hannelore Heidemann, Michael Heise, Matthias Hesse, Ercan Kertmen, Yüksel König, Ernst Kraas, Colin M. Krüger, Rainer Kube, Stefan Kürbis, Stefan Lenz, Martin Loss, Frank Marusch, Mario Müller, Arnd Müller, Carolin Oeder, Gero Puhl, Thomas Rost, Markus Scheibel, Roland Scherer, Moritz Schmelzle, Thomas Steinmüller, Ulrich Stöckle, Sophie Strozyk, Henryk Thielemann, Heinz-Peter Vetter, Arndt von Kirchbach, Sascha Weiß

**Affiliations:** 1 Berlin-Brandenburgische Chirurgische Gesellschaft, Berlin, Deutschland; 2 Chirurgische Klinik, Charité – Universitätsmedizin Berlin, Corporate Member of Freie Universität Berlin and Humboldt-Universität zu Berlin, Campus Charité Mitte und Campus Virchow Klinikum, Augustenburger Platz 1, 13353 Berlin, Deutschland

**Keywords:** Neuhardenberger Gespräche, Positionspapier, Arbeitszeitmodelle, Spezialisierung, Weiterbildungsverbände, Chirurgische Weiterbildung, Neuhardenberg talks, Position paper, Working hours models, Specialization, Further training associations, Surgical further training

## Abstract

**Hintergrund:**

Die chirurgische Weiterbildung steht vor der anspruchsvollen Aufgabe, technische Fortschritte und Patientensicherheit in Einklang zu bringen, insbesondere im Kontext der geplanten Krankenhausreform. Zusätzlich stellen der Generationswechsel und veränderte Ansprüche der Generationen Y und Z an den Arbeitsplatz weitere Herausforderungen dar. Um diesen Anforderungen gerecht zu werden, hat die Berlin-Brandenburgische Chirurgische Gesellschaft (BCG) während der „Neuhardenberger Gespräche“ eine strukturierte Diskussion initiiert und ein Positionspapier erarbeitet.

**Methodik:**

Im Rahmen der Neuhardenberger Gespräche fanden vier Sitzungen mit Impulsvorträgen und Diskussionen statt. Auf Grundlage der Hauptdiskussionspunkte wurden anschließend Thesen und Positionen erstellt, über die digital abgestimmt wurde.

**Ergebnisse:**

Die Resultate zeigen einen klaren Konsens für flexible Arbeitszeitmodelle, frühere Spezialisierungsmöglichkeiten und die Integration externer Rotationen in die chirurgische Weiterbildung. In Bezug auf Talentakquisition und Frührekrutierung von Weiterbildungsassistent*innen zeigte sich ein eindeutiger Konsens für die Förderung von Mitarbeiterengagement und strukturierte Frührekrutierung von Studierenden. Es herrschte Einigkeit hinsichtlich der Einführung von Weiterbildungsverbänden als wirksames Mittel, um eine hochwertige chirurgische Weiterbildung zu gewährleisten.

**Diskussion:**

Einer der zentralen Punkt in den Diskussionen war, dass eine hochwertige chirurgische Weiterbildung nur in Weiterbildungsverbänden realisierbar sein wird, insbesondere vor dem Hintergrund der anstehende Krankenhausreform. Die BCG plant die Ausarbeitung eines modularen Weiterbildungsverbands, um die chirurgische Weiterbildung in Berlin/Brandenburg zukunftsfähig zu gestalten.

**Zusatzmaterial online:**

Zusätzliche Informationen sind in der Online-Version dieses Artikels (10.1007/s00104-024-02071-4) enthalten.

## Abstract (en)

**Background:**

Surgical further training faces the challenging task of reconciling technological advancements and patient safety, particularly in the context of the planned hospital reform. Additionally, the generation shift and evolving expectations of Generations Y and Z in the workplace present further challenges. In response to these demands, the Berlin-Brandenburg Surgical Society (Berlin-Brandenburgische Chirurgische Gesellschaft, BCG) initiated a structured discussion and developed a position paper during the Neuhardenberg talks (Neuhardenberger Gespräche).

**Methodology:**

Within the framework of the Neuhardenberg talks, four sessions with keynote presentations and discussions took place. Based on the main discussion points, theses and positions were subsequently formulated and digitally voted on.

**Results:**

The results reveal a clear consensus favoring flexible working hours models, earlier specialization options and the integration of external rotations in surgical further training. Regarding talent acquisition and early recruitment of residents, there was a clear consensus supporting the promotion of employee engagement and structured early recruitment of students. There was unanimous agreement on the introduction of training associations as an effective means to ensure high-quality surgical further training.

**Discussion:**

One of the central points in the discussions was that high-quality surgical further training will only be achievable within training associations, especially given the impending hospital reform. The BCG plans to develop a modular further training association to make surgical further training in Berlin/Brandenburg fit for the future.

## Hintergrund

Die chirurgische Weiterbildung stand stets vor der Herausforderung, technische Fortschritte und die Patientensicherheit in Einklang zu bringen. Mit zunehmender Arbeitsbelastung und dem wachsenden wirtschaftlichen Druck im Gesundheitssystem kommen weitere Faktoren hinzu. Um auch in diesem sich rasch verändernden Umfeld eine qualitativ hochwertige chirurgische Weiterbildung sicherzustellen, sind effektive strukturelle Veränderungen erforderlich. Insbesondere unter Berücksichtigung der geplanten Krankenhausreform müssen diese Anpassungen durchgeführt werden, um die Zukunftsfähigkeit der chirurgischen Weiterbildung und somit des chirurgischen Fachs in Deutschland zu gewährleisten [1]. Die von der Regierungskommission für eine moderne und bedarfsgerechte Krankenhausversorgung angestrebte bundesweite Gradierung der Krankenversorgung, seien es nun Leistungsgruppen oder Versorgungslevel, wird vor allem für Krankenhäuser der Grund- und Regelversorgung Einschnitte in der chirurgischen Weiterbildung mit sich bringen.

Zusätzlich stellt der unaufhaltbar voranschreitende Generationswechsel die chirurgischen Disziplinen vor weitere große Herausforderungen.

Mehrere Umfragen haben gezeigt, dass zu Beginn des Medizinstudiums ein starkes Interesse an einer chirurgischen Weiterbildung besteht, dieses jedoch zum Ende des Studiums deutlich abnimmt [2, 3]. Die chirurgischen Disziplinen müssen sich somit selbstkritisch hinterfragen, wie man diesem Trend entgegenwirken und bereits Studierende mit nachhaltigen Angeboten für eine chirurgische Weiterbildung gewinnen kann.

Ein weiterer wichtiger Aspekt, der von den chirurgischen Disziplinen hinsichtlich der Weiterbildung und Mitarbeiterakquise berücksichtig werden muss, ist der sich wandelnde Anspruch der künftigen Generation an einen potenziellen Arbeitgeber. Die künftigen chirurgischen Weiterbildungsassistent*innen gehören den Generationen Y und Z an, deren Ansprüche an einen Arbeitsplatz sich deutlich von denen der vorangegangenen Generationen unterscheiden. So hat eine Umfrage für die Generation Y von Kasch et al. ergeben, dass für den Nachwuchs Faktoren wie Work-Life-Balance, Karriere, fachlicher Anspruch und Betriebsklima von entscheidender Bedeutung sind [4]. Die Jahrgänge der Generation Z suchen Sicherheit und Stabilität, eine klare Trennung von Berufs- und Privatleben, aber auch mehr Mitspracherecht [5].

Eine qualitativ hochwertige und strukturierte Weiterbildung unter geregelten Arbeitsbedingungen vermag künftig einer der wichtigsten Treiber der Arbeitsplatzzufriedenheit sein. Aufgrund des Nachwuchsmangels stellen mittlerweile Bewerber*innen Anforderungen an ihren künftigen Arbeitsplatz, während Klinikleitungen bereits seit längerem nicht mehr aus einem großen Pool von Bewerber*innen auswählen können [3].

Vor dem Hintergrund des demographischen Wandels ist das Gesundheitswesen nicht nur mit einem steigenden Bedarf an medizinischer Versorgung konfrontiert, sondern auch mit einer alternde Ärzteschaft, die den Fachkräftemangel zusätzlich unterstützt [3].

Um sich diesem Spannungsfeld zu stellen, hat sich die Berlin-Brandenburgische Chirurgische Gesellschaft (BCG) in Rahmen eines *Retreats *(„*Neuhardenberger Gespräche*“) mit dem Thema der Weiterbildung in den chirurgischen Disziplinen und Akquise/Rekrutierung des chirurgischen Nachwuchses auseinandergesetzt.

## Methodik

Im Rahmen der Neuhardenberger Gespräche wurden vier Sitzungen mit mehreren, unterschiedlich gefächerten Impulsvorträgen organisiert (Programm und Vortragsthemen, Anhang 2). Die Vorträge dienten jeweils als Diskussionsanstoß für die Teilnehmer*innen, strukturiert und geleitet wurde die Diskussion von zwei Sitzungsvorsitzenden. Im Rahmen der Diskussion wurden Thesen und Positionen erarbeitet, über welche nach Abschluss jeder Sitzung mittels einer anonymen QR-Code-basierte Abstimmung über *Google Forms *abgestimmt wurde (Google LLC, Mountain View, CA, Vereinigten Staaten von Amerika).

Für die Erstellung des Positionspapiers wurden nur die Statements genutzt, welche im Zusammenhang mit Weiterbildung und Talentakquise standen, alle anderen Statements und Abstimmungsergebnisse finden sich im Anhang 3.

Wir definierten Konsensstärke hinsichtlich eines Statements wie folgt:

1. Starker Konsens > 95 % Zustimmung
2. Konsens > 75–95 %
3. Mehrheitliche Zustimmung > 50–75 %
4. Dissens < 50 % [6].

## Ergebnisse und Diskussion

### Sitzung 1 – flächendeckende Weiterbildung und Weiterbildungskooperation (I)

**Abb. 1:**
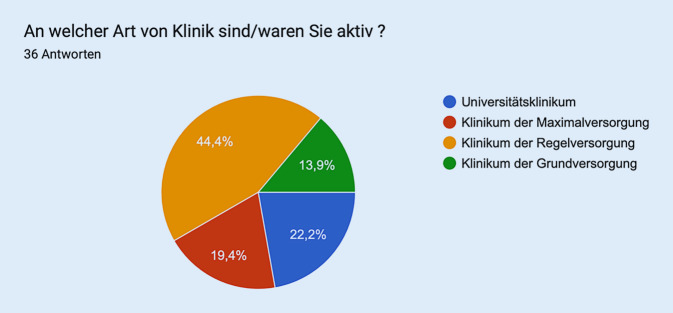
Aufteilung der Abstimmenden (*n* = 36) in Sitzung 1 – flächendeckende Weiterbildung und Weiterbildungskooperation

Die Aufteilung der Abstimmenden ist Abb. 1 zu entnehmen.

*Es sollten vermehrt flexible Arbeitszeitmodelle in der Chirurgie ermöglicht werden und in der Weiterbildungsordnung berücksichtigt werden.*

- *Zustimmung: 91,7* *% (n* *=* *33)*
- *Ablehnung: 8,3* *% (n* *=* *3)*
- *Konsensstärke: Konsens*

In einer Umfrage des BDC zeigten sich fast zwei Drittel der Befragten unzufrieden mit den Arbeitsbedingungen. Zusätzlich sagten nur knapp etwas mehr als 50 % der Befragten, dass auf die Einhaltung des Arbeitszeitgesetzes geachtet wird [7].

Um dieser Unzufriedenheit entgegenzuwirken, mehr Konformität mit dem Arbeitszeitgesetz zu erzielen und den Beruf für Weiterbildungsassistent*innen attraktiver zu machen, ist die Implementierung flexibler, moderner Arbeitszeitmodelle in der Chirurgie unausweichlich.

*In der chirurgischen Weiterbildung sollte früher eine Wahl zwischen Spezialisierung oder Generalisierung ermöglicht werden.*

- *Zustimmung: 61,1* *% (n* *=* *22)*
- *Ablehnung: 38,9* *% (n* *=* *14)*
- *Konsensstärke: mehrheitliche Zustimmung*

Diese Thematik wurde kontrovers diskutiert. Die zunehmende Spezialisierung in der Chirurgie ist sicherlich der rasanten technisch-chirurgischen Weiterbildung geschuldet. Auch die zunehmende Evidenz für die Steigerung der Qualität durch eine Spezialisierung treibt diese voran. Jedoch wird die zunehmende Spezialisierung auch von zahlreichen Chirurg*innen kritisch gesehen, was sich beispielsweise in der Gründung eines Vereines „Generalisten in der Chirurgie e. V.“ äußert [8].

### Sitzung 2 – Weiterbildungskooperation (II) und Personalrekrutierung

**Abb. 2:**
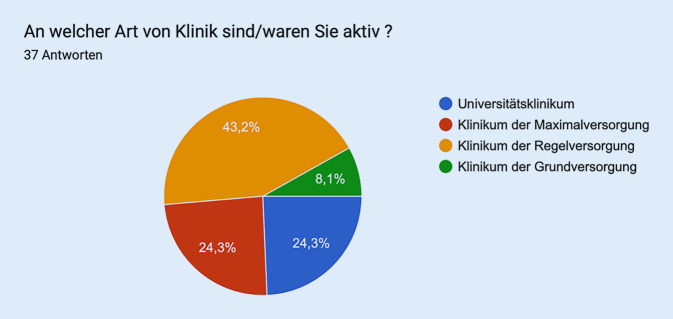
Aufteilung der Abstimmenden (*n* = 37) in Sitzung 2 – Weiterbildungskooperation und Personalrekrutierung

Die Aufteilung der Abstimmenden ist Abb. 2 zu entnehmen.

*Es sollte eine Integration definierter externer Rotationen in die chirurgische Weiterbildung erfolgen.*

- *Zustimmung: 94,6* *% (n* *=* *35)*
- *Ablehnung: 5,4* *% (n* *=* *2)*
- *Konsensstärke: Konsens*

Bezüglich der Integration definierter externer Rotationen herrschte große Einigkeit. Vor allem vor dem Hintergrund der dritten Stellungnahme und Empfehlung der Regierungskommission für eine moderne und bedarfsgerechte Krankenhausversorgung und dem darin dargelegten Versorgungsstufenmodell werden externe Rotationen und dadurch bedingte Kooperationen unabdingbar. Durch die geplante Umstrukturierung wird aus Sicht der BCG eine komplette chirurgische Weiterbildung an einem Krankenhaus nicht mehr flächendeckend möglich sein.

Es sollte eine Integration von Fellowships (Facharzt*innenniveau) in die chirurgische Weiterbildung erfolgen.

- *Zustimmung: 89,2* *% (n* *=* *33)*
- *Ablehnung: 10,8* *% (n* *=* *4)*
- *Konsensstärke: Konsens*

Fellowships sind vor allem im angloamerikanischen Raum nach der chirurgischen Grundweiterbildung ein viel genutztes Mittel zur weiteren Spezialisierung. Im deutschsprachigen Raum sind Fellowships im klassischen Sinne bisher eine Rarität. Dennoch wurde in unserer Umfrage die Implementierung befürwortet. Hierbei muss jedoch berücksichtigt werden, dass die Integration von Fellowships mitunter die Qualität der chirurgischen Grundweiterbildung (bis zum/zur Fachärzt*in) reduziert. So konnte in einem großen Survey in Australien und Neuseeland erhoben werden, dass Assistent*innen in Kliniken ohne Fellows operativ in mehr Major- und Minor-Fälle involviert waren [9].

*Die Eingriffszahlen in den Weiterbildungskatalogen der chirurgischen Disziplinen sollte reduziert werden.*

- *Zustimmung: 59,5* *% (n* *=* *22)*
- *Ablehnung: 40,5* *% (n* *=* *15)*
- *Konsensstärke: mehrheitliche Zustimmung*

Die Novellierung der Weiterbildungsordnung hin zu einer kompetenzbasierten Weiterbildung lässt in den chirurgischen Disziplinen die Frage aufkommen, wie oft ein Eingriff unter Supervision durchgeführt werden muss, bis dieser als Fachärzt*in eigenverantwortlich durchgeführt werden kann.

*Die Assistenz durch die Weiterbildungsbefugten sollte in der chirurgischen Weiterbildung einen größeren Stellwert einnehmen.*

- *Zustimmung: 89,2* *% (n* *=* *33)*
- *Ablehnung: 10,8* *% (n* *=* *4)*
- *Konsensstärke: Konsens*

Das Erlernen operativer Fähigkeiten ist der wichtigste Punkt in der chirurgischen Weiterbildung, welches mitunter mit einer Verlängerung der Operationszeit vergesellschaftet [10]. Vor dem Hintergrund des Ökonomisierungdrucks und der nicht im DRG-System abgebildeten Kosten für chirurgische Weiterbildung führt diese Konstellation in vielen Kliniken zu großen Problemen.

Es sollte strukturierte Integrationsprogamme für chirurgische Weiterbildungsassistent*innen geben.

- *Zustimmung: 94,6* *% (n* *=* *35)*
- *Ablehnung: 5,4* *% (n* *=* *2)*
- *Konsensstärke: Konsens*

Der Nachwuchsmangel in der Chirurgie führt dazu, dass in strukturschwächeren Regionen freie Stellen mit außerhalb von Deutschland ausgebildeten, europäischen und außereuropäischen Ärzt*innen besetzt werden [11]. Um hier weiterhin eine qualitativ hochwertige Patientenversorgung, aber auch chirurgische Weiterbildung zu gewährleisten, ist die Implementierung von Integrationsmaßnahmen von großer Bedeutung [11].

*Es sollte differenzierte Weiterbildungsordnungen für unterschiedliche Versorgungslevels geben.*

- *Zustimmung: 64,9* *% (n* *=* *24)*
- *Ablehnung: 35,1* *% (n* *=* *13)*
- *Konsensstärke: mehrheitliche Zustimmung*

Hinsichtlich dieser These war der Konsens unter den Teilnehmenden weniger stark. Abgesehen von dem wahrscheinlich schwierig umzusetzenden Rechtsrahmen wurde bei der Diskussion kontrovers über die Definition solcher differenzierter Weiterbildungsordnungen diskutiert.

*Es soll für Weiterbildungsbefugte ein professionelles Leadership-Training geben.*

- *Zustimmung: 83,8* *% (n* *=* *31)*
- *Ablehnung: 16,2* *% (n* *=* *6)*
- *Konsensstärke: Konsens*

In der Wirtschaft sind Leadership-Trainings ein viel genutztes Tool, in welches die Unternehmen viel Geld investieren [12]. Jedoch muss der Nutzen solcher Trainings auch kritisch hinterfragt werden [12].

Es muss festgehalten werden, dass eine moderne professionelle Führung einer Klinik als eine der wichtigsten Säulen des Personalmanagements angesehen werden kann und vor dem Hintergrund des Fachkräftemangels von essenzieller Bedeutung ist [13]. Grade und Ghadimi haben in einem Leitthema in *Die Chirurgie *konstatiert, dass die Chirurgie es schwer haben wird „ohne moderne und nachhaltige Konzepte im Personalmanagement“ [13].

### Sitzung 3 – Talentakquisition und Frührekrutierung

**Abb. 3:**
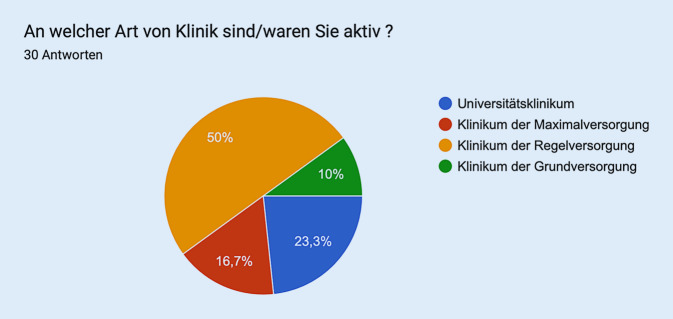
Aufteilung der Abstimmenden (*n* = 30) in Sitzung 3 – Talentakquisition und Frührekrutierung

Die Aufteilung der Abstimmenden ist Abb. 3 zu entnehmen.

*Mitarbeiterengagement sollte in der Chirurgie ein größerer Stellenwert eingeräumt werden.*

- *Zustimmung: 100* *% (n* *=* *30)*
- *Ablehnung: 0* *% (n* *=* *0)*
- *Konsensstärke: starker Konsens*

*Mitarbeiterengagement ist in den chirurgischen Disziplinen umsetzbar.*

- *Zustimmung: 100* *% (n* *=* *30)*
- *Ablehnung: 0* *% (n* *=* *0)*
- *Konsensstärke: starker Konsens*

Bezüglich dieser beiden Statements herrschte ein starker Konsens. Es ist bekannt, dass Mitarbeiterengagement essenziell für den Erfolg eines Unternehmens ist [14]. Auch in der Chirurgie wurde das Mitarbeiterengagement mittlerweile als ein wichtiger Faktor erkannt [13]. Jedoch besteht hinsichtlich der flächendeckenden Ausgestaltung noch Handlungsbedarf.

*Eine Frührekrutierung von Studierenden für die chirurgischen Disziplinen sollte strukturiert implementiert werden.*

- *Zustimmung: 90* *% (n* *=* *27)*
- *Ablehnung: 10* *% (n* *=* *3)*
- *Konsensstärke: Konsens*

Dieses Statement ist unter Berücksichtigung der in der Einleitung erwähnten Studien zu sehen, welche gezeigt haben, dass das Interesse an chirurgischen Weiterbildungen während des Studiums abnimmt. Hier müssen die chirurgischen Disziplinen intervenieren und mit niedrigschwelligen Angeboten (z. B. Studierendenkongresse oder Workshops) frühzeitig im Studium das Fach attraktiv repräsentieren, um für eine chirurgische Weiterbildung zu werben [13]. Es bleibt zusätzlich abzuwarten, welchen Einfluss die Umstrukturierung des Medizinstudiums hin zu sog. Modellstudiengängen hat, welche mit einer engeren Verknüpfung von Klinik und Vorklinik einhergehen.

Auch nichtuniversitäre Chirurg*innen sollte sich in die Frührekrutierung von Studierenden einbringen.

- *Zustimmung: 93,3* *% (n* *=* *27)*
- *Ablehnung: 6,7* *% (n* *=* *2)*
- *Konsensstärke: Konsens*

Der bereits vielfach erwähnte Nachwuchsmangel in den chirurgischen Disziplinen stellt viele Kliniken vor allem im ländlichen Raum vor die Herausforderung, ausreichend Weiterbildungsassistent*innen zu finden, um eine qualitative hochwertige Patientenversorgung zu gewährleisten. Wie die Umfrage von Kasch et al. bereits zeigte, sind Karriere und Prestige wichtige Faktoren für die Arbeitsplatzwahl [4]. Bei diesen Punkten haben Universitätskliniken sicherlich einen gewissen Vorteil. Um jedoch flächendeckend mehr chirurgische Weiterbildungsassistent*innen zu gewinnen, sollten sich aus Sicht der Teilnehmenden auch nichtuniversitäre Chirurg*innen mehr in die Frührekrutierung einbringen.

### Sitzung 4 – Moderne Weiterbildungskonzepte und Weiterbildungsverbände

**Abb. 4:**
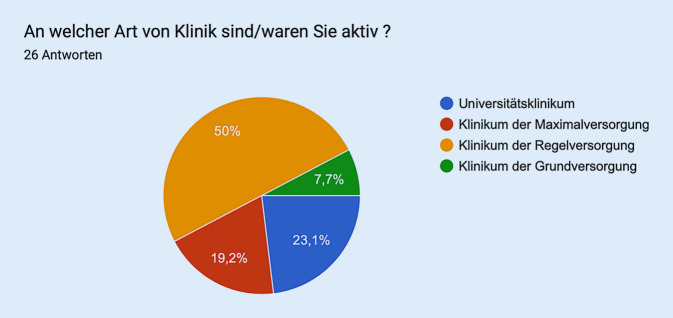
Aufteilung der Abstimmenden (*n* = 26) in Sitzung 4 – Moderne Weiterbildungskonzepte und Weiterbildungsverbände

Die Aufteilung der Abstimmenden ist Abb. 4 zu entnehmen.

*Die Wirtschaftlichkeit steht im Widerspruch zur chirurgischen Weiterbildung.*

- *Zustimmung: 80,8* *% (n* *=* *21)*
- *Ablehnung: 19,2* *% (n* *=* *5)*
- *Konsensstärke: Konsens*

Wie bereits erwähnt, ist der ökonomische Druck im Medizinsektor enorm. Unter Berücksichtigung der Rahmenbedingungen (u. a. kurze Operationszeiten und Liegedauern sowie Arbeitsverdichtung) ist die Qualität der chirurgischen Weiterbildung in Gefahr.

*In der Weiterbildung sollte berücksichtigt werden, dass die Qualität der Patientenversorgung oberste Priorität hat und sich dieser einordnet.*

- *Zustimmung: 96,2* *% (n* *=* *25)*
- *Ablehnung: 3,8* *% (n* *=* *1)*
- *Konsensstärke: starker Konsens*

Die Qualität der Patientenversorgung ist das höchste Gut der ärztlichen Tätigkeit. Die chirurgischen „Lehrer*innen“ befinden sich immer im Spannungsfeld zwischen chirurgischer Weiterbildung und Qualität der Patientenversorgung bzw. Patientensicherheit [15].

*Weiterbildungsverbände sind ein geeignetes Tool, um flächendeckend qualitativ hochwertige Weiterbildung anzubieten.*

- *Zustimmung: 100* *% (n* *=* *26)*
- *Ablehnung: 0* *% (n* *=* *0)*
- *Konsensstärke: starker Konsens*

*Die BCG wird sich dafür einsetzen, diese Weiterbildungsverbände zu implementieren.*

- *Zustimmung: 100* *% (n* *=* *26)*
- *Ablehnung: 0* *% (n* *=* *0)*
- *Konsensstärke: starker Konsens*

*Die BCG soll eine Projektgruppe für die Einrichtung von Weiterbildungsverbänden ins Leben rufen.*

- *Zustimmung: 100* *% (n* *=* *26)*
- *Ablehnung: 0* *% (n* *=* *0)*
- *Konsensstärke: starker Konsens*

*Die BCG sollte sich dafür einsetzen, dass technische/organisatorische Plattformen in Rahmen von Kooperationsverbänden etabliert werden.*

- *Zustimmung: 100* *% (n* *=* *26)*
- *Ablehnung: 0* *% (n* *=* *0)*
- *Konsensstärke: starker Konsens*

*Die Rahmenbedingungen für die Weiterbildungsverbände sollen gemeinsam zwischen der BCG und der Berliner/Bran-denburger Ärztekammer definiert werden.*

- *Zustimmung: 100* *% (n* *=* *26)*
- *Ablehnung: 0* *% (n* *=* *0)*
- *Konsensstärke: starker Konsens*

Im Rahmen der Neuhardenberger Gespräche wurden, basierend auf einem Impulsvortrag von Herrn Professor Dr. med. Johann Pratschke (Charité Universitätsmedizin Berlin), Weiterbildungsverbände als ein geeignetes Mittel propagiert, um eine zukunftsfähige, qualitativ hochwertige chirurgische Weiterbildung im Raum Berlin/Brandenburg unter den sich rapide ändernden Rahmenbedingungen zu gewährleisten. Diese Weiterbildungsverbände sollen basierend auf definierten Weiterbildungscurricula die gesamten Inhalte der Weiterbildungsordnung abdecken. Durch die geplante Umstrukturierung im Rahmen der Krankenhausreform wird es zwangsläufig zur Umstrukturierung chirurgischer Kliniken kommen, welche eine Adaptation der chirurgischen Weiterbildung mit sich bringen wird. Durch die Implementierung von Weiterbildungsverbänden wird den Weiterbildungsassistent*innen, unabhängig vom später geplanten Arbeitsziel, die Möglichkeit gegeben, eine vielschichtige, qualitativ hochwertige Weiterbildung zu genießen. Um die Ausgestaltung dieser Weiterbildungsverbände voranzutreiben, haben sich die Teilnehmenden dafür mit einem starken Konsens ausgesprochen, eine Projektgruppe zu initiieren, die sich engmaschig mit den Ärztekammern Berlin/Brandenburg abstimmt, um die technischen und organisatorischen Vorrausetzungen für solche Weiterbildungsverbände zu definieren und zu strukturieren.

## Diskussion

Während der Weiterbildung muss der/die angehende Chirurg*in seine/ihre operativen Fähigkeiten kontinuierlich weiterentwickeln und schrittweise perfektionieren [16].

Die Aufgabe der chirurgischen Lehrer*innen ist es, die operative Expertise in den Weiterbildungsassistent*innen zu etablieren und weiterzuentwickeln. So können die Lehrenden von chirurgischen Techniken eine chirurgische Schule/Tradition etablieren und stehen zugleich mit ihren Schüler*innen in einer gegenseitigen Wechselbeziehung [17]. Jedoch hat sich aufgrund der immer komplexeren chirurgischen Techniken das Verständnis einer chirurgischen Schule gewandelt. So wird das Wissen heutzutage nicht mehr nur von einem/einer chirurgischen Lehrer*in vermittelt.

Obwohl in Deutschland die Weiterbildungsordnung novelliert wurde, hin zu einer kompetenzbasierten Weiterbildung, spielen in den chirurgischen Disziplinen Eingriffszahlen weiterhin eine wichtige Rolle. Es braucht eine gewissen Anzahl des jeweiligen Eingriffes, um die manuellen und technischen Fähigkeiten zu erlernen und diese eigenständig und ohne Supervision durchführen zu können. Trotz der klaren Anforderungen, die in der Weiterbildungsordnung formuliert sind, haben Umfragen gezeigt, dass die chirurgische Weiterbildung in Deutschland teilweise als unstrukturiert empfunden wird [18, 19]. Dieser Fakt ist sicherlich ein Treiber des in der Einleitung bereits erläuterten Nachwuchsmangels. Überspitzt formuliert könnte man sagen, dass die Struktur und Qualität der Weiterbildung auch über die Chirurgie der Zukunft entscheiden wird.

Die Weiterbildungskonzepte und Strukturen in der Chirurgie unterscheiden sich international deutlich [19]. So haben Kanada und die Schweiz sehr strukturierte Curricula [19]. Außerdem gibt es beispielsweise in der Schweiz reguläre Zwischenprüfungen, wobei auch in diesen strukturierten Curricula insgesamt wenig Arbeitszeit explizit für Weiterbildung genutzt werden kann [19].

Wie bereits erwähnt ist aus unserer Sicht die zunehmende Arbeitsverdichtung und Ökonomisierung eine wichtige Größe, die die Art und Qualität der Weiterbildung stark negativ beeinflusst.

Die Ergebnisse der Umfrage bei den Neuhardenberger Gesprächen zeigt ganz klar, dass die Probleme in der chirurgischen Weiterbildung von der „*Community*“ erkannt worden sind und ein starker Konsens besteht, diese Probleme gemeinsam strukturiert und forciert zu adressieren. Hier haben die Teilnehmenden bereits reichlich spannende und vielversprechende Ansatzpunkte erarbeitet und formuliert. Hier hat sich als wichtigster Punkt die Bildung von Weiterbildungsverbänden herauskristallisiert.

Aus unserer Sicht sollte das Curriculum eines solchen Weiterbildungsverbandes einen modularen Aufbau haben. Zur Formierung des Weiterbildungsverbandes müsste erhoben werden, welche weiterbildungsrelevanten Eingriffe und Inhalte in der jeweiligen Klinik vermittelt werden können. Da unter Berücksichtigung der weiteren Etablierung von Mindestmengen und der geplanten Krankenhausreform nicht mehr alle Kliniken die geforderten Inhalte der Weiterbildungsordnung abbilden werden können. Dies wird in den jeweiligen Kliniken zu einer weiteren Fokussierung, respektive Spezialisierung führen. Dies wird einen modularen/spezialisierten Curriculumsaufbau unabdingbar machen.

**Abb. 5:**
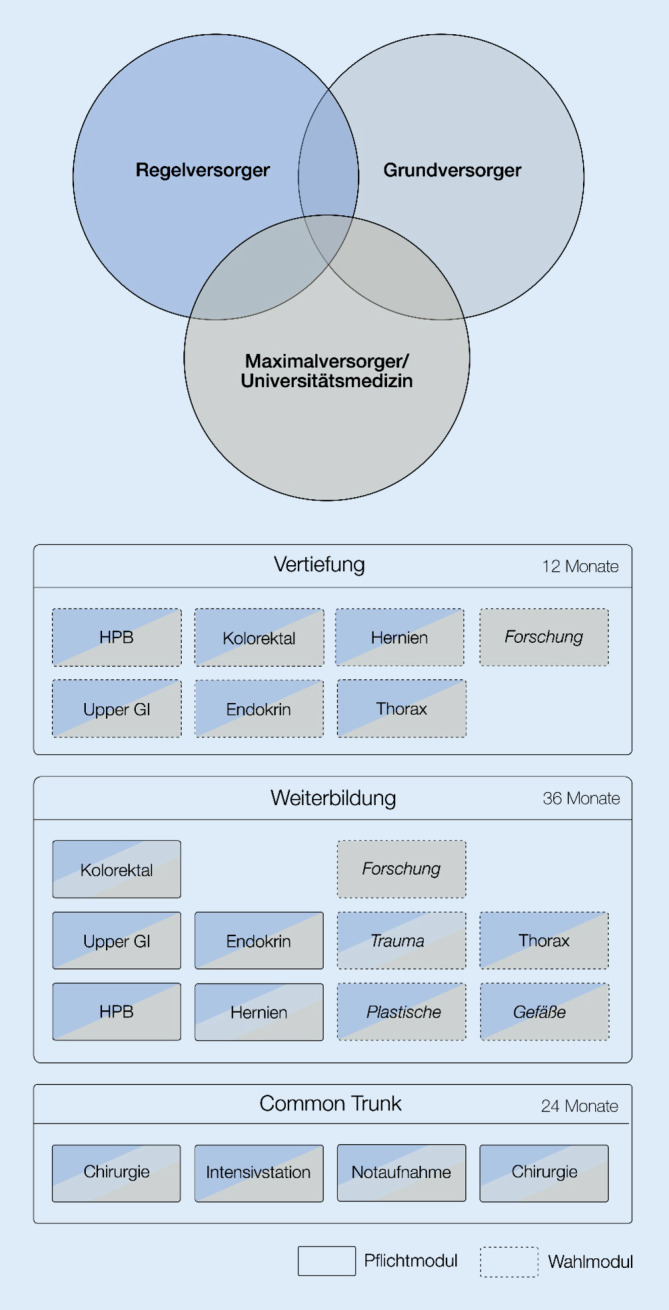
Modulares Weiterbildungscurriculum eines Weiterbildungsverbandes. *HPB *hepatopankreatobiliär,* Upper GI *oberer Gastrointestinaltrakt

Ein möglicher Ablauf eines solchen modularen Weiterbildungscurriculums ist in Abb. 5 dargestellt. Dieser modulare Aufbau, mit einem strukturierten Rotationskonzept hat in vielerlei Hinsicht Vorteile. Hier wäre unter anderem zu nennen, dass durch die oben angesprochen Fokussierungen/Spezialsierungen die chirurgische Weiterbildung in den jeweiligen Modulen deutlich präziser und strukturierter durchgeführt werden wird. Langfristig wird sich dies sicherlich auch positiv auf die ärztliche Patientenversorgung auswirken. Des Weiteren würde der modulare Aufbau zu deutlich mehr Strukturierung in der Weiterbildung führen und die oft bemängelte fehlende Struktur in der Weiterbildung adressieren. Dies führt hoffentlich dazu, dass sich gerade aus der Generation Y potenzielle Weiterzubildende für eine Laufbahn in der Chirurgie entscheiden werden.

Am Ende des von uns vorgeschlagenen modularen Weiterbildungscurriculums steht aus unserer Sicht ein/e exzellent weitergebildet/e Chirurg*in, welche/r die Fähigkeiten hat, selbständig Eingriffe der kompetenzbasierten Weiterbildungsordnung durchzuführen.

Die genaue Ausgestaltung der modulären Weiterbildungsverbände muss im Einzelnen noch erarbeitet werden und die rechtlichen und strukturellen Rahmenbedingungen geklärt werden. Hierfür hat die BCG eine Projektgruppe „chirurgische Weiterbildung“ ins Leben gerufen, welche die konzeptionelle Ausarbeitung eines modulären Weiterbildungsverbandes in der Metropolregion Berlin/Brandenburg in Angriff nehmen wird. Hierbei werden ebenfalls die geographischen und strukturellen Besonderheiten der Hauptstadtregion berücksichtigt werden. Die BCG hofft, dass dieses Positionspapier als ein Impulsgeber fungiert, um in Deutschland die chirurgische Weiterbildung zukunftsfähig zu machen.

## Zusammenfassung und Schlussfolgerung

Die BCG hat sich bei den Neuhardenberger Gesprächen mit den wichtigsten Themenkomplexen bezüglich einer zukunftsfähigen und modernen chirurgischen Weiterbildung sowie der Akquisition junger, motivierter Weiterbildungsassistent*innen auseinandergesetzt. Für die BCG steht fest, dass eine qualitativ herausragende Weiterbildung, vor allem vor dem Hintergrund der geplanten Krankenhausreform, zahlreiche Modifizierungen benötigt. Als geeignetstes Tool erscheinen hierfür Weiterbildungsverbände mit definierten Curricula, welche alle in der Weiterbildungsordnung geforderten Inhalte abbilden. Mit dieser Maßnahme könnte unter den sich ändernden Rahmenbedingung eine flächendeckende, qualitativ hochwertige chirurgische Weiterbildung gewährleistet werden.

Um diese Anforderungen an die chirurgische Weiterbildung für den Raum Berlin/Brandenburg zu adressieren, hat die BCG eine Projektgruppe „Chirurgische Weiterbildung“ initiiert, die sich mit den Rahmenbedingungen für Weiterbildungsverbände auseinandersetzen wird.

Diese Rahmenbedingungen für Weiterbildungsverbände werden in enger Abstimmung mit den verantwortlichen Ärztekammern getroffen werden, welche ebenfalls an der Projektgruppe beteiligt sind.

### Supplementary Information

Anhang
