# Supplementary material for: Position paper of the Berlin-Brandenburg Surgical Society—On the future of surgical further training
Source: Chirurgie (Heidelb). 2024 Apr 24;95(7):555–62. [Article in German] doi: 10.1007/s00104-024-02071-4 (PMC11190009; doi:10.1007/s00104-024-02071-4)

Anhang 1 Teilnehmende Neuhardenberger Gespräche

Vorname	Nachname
Ulrich	Adam
Georg	Bauer
Christoph	Benckert
Mike	Bereuter
Katharina	Beyer
Peter	Bobbert
Joachim	Böttger
Yao	Chen
Lope	Estévez Schwarz
Stefan	Farke
Ullrich	Fleck
Marek	Frakowiak
Georg	Fritzsch
Klaus	Gellert
Hannelore	Heidemann
Michael	Heise
Matthias	Hesse
Ercan	Kertmen
Yüksel	König
Ernst	Kraas
Colin M.	Krüger
Rainer	Kube
Stefan	Kürbis
Stefan	Lenz
Martin	Loss
Frank	Marusch
Mario	Müller
Arnd	Müller
Carolin	Oeder
Gero	Puhl
Thomas	Rost
Markus	Scheibel
Roland	Scherer
Moritz	Schmelzle
Thomas	Steinmüller
Ulrich	Stöckle
Sophie	Strozyk
Henryk	Thielemann
Heinz-Peter	Vetter
Arndt	von Kirchbach
Sascha	Weiß

Anhang 2 – Programm Neuhardenberger Gespräche

Berliner Chirurgische Gesellschaft

Vereinigung der Chirurgen

Berlins und Brandenburgs

Programm - Neuhardenberger Gespräche

Herzlich Willkommen bei den Neuhardenberger Gesprächen!

Neben der Frage, wie chirurgische Weiterbildung im ökonomischen Spannungsfeld möglich ist und erfolgen kann, drängt sich heutzutage zusätzlich die Frage des aktuellen Zeitgeistes und des Mindsets der jungen Kolleginnen und Kollegen auf. Die Etablierung von Weiterbildungskooperationen, moderne Weiterbildungskonzepte aber auch Nachwuchsförderung und Mitarbeiterbindung vor dem Hintergrund des demographischen Wandels sind aktuelle Themen der modernen Chirurgie.

Das Programm beinhaltet mehrere Impulsvorträge mit entsprechender Zeit für angeregte und offene Diskussionen. Schloss Neuhardenberg soll abseits des klinischen Alltages eine entspannte Atmosphäre bieten, um dringende Themen im kleinen Kreis intensiv zu diskutieren, die verschiedenen Perspektiven und Problematiken der einzelnen Krankenhäuser zu beleuchten und gemeinsam Lösungsstrategien zu erarbeiten, um die Zukunft unseres Faches Chirurgie weiterhin attraktiv zu gestalten.

Im Namen der Berliner Chirurgischen Gesellschaft möchten wir Sie herzlich begrüßen,

Ihr

Univ.-Prof. Dr. med. J. Pratschke

Kontaktdaten:
Prof. Dr. med. J. Pratschke (johann.pratschke@charite.de)
PD Dr. med. F. Krenzien – Tagungssekretär (felix.krenzien@charite.de)
PD Dr. med. E. Dobrindt – Tagungssekretärin (eva-maria.dobrindt@charite.de)

Buchung der Zimmer unter: 033476 600-0, Stichwort „BCG-Neuhardenberger Gespräche“ bis zum 31.02.2023
(Übernachtungskosten sind selbst zu tragen)

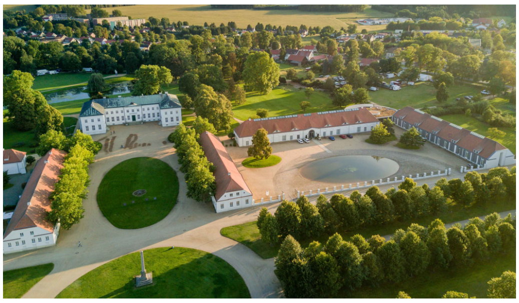

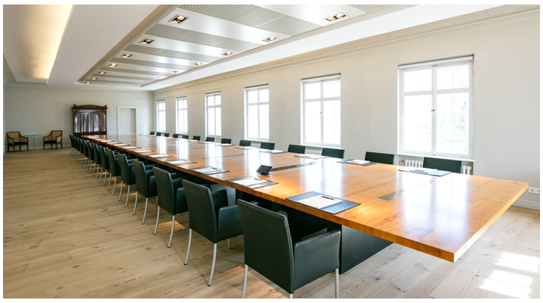

Schloss Neuhardenberg, Schinkelplatz 1-8, 15320 Neuhardenberg

1. Tag: Freitag, 03. März 2023

13.00 Uhr **Come together**

Chair: Johann Pratschke und Yüksel König
(Ort: Großer Saal, Schloss Neuhardenburg)

14:00 Uhr **Wie kann die chirurgische Weiterbildung flächendeckend gewährleistet werden**
Peter Bobbert, Berliner Ärztekammer
(30 min Vortrag + 30 min Diskussion + Abstimmung über Thesen)

15:00 Uhr **Weiterbildungskooperation aus Sicht eines Maximalversorgers**
Rainer Kube, Carl-Thiem-Klinikum Cottbus
(30 min Vortrag + 30 min Diskussion + Abstimmung über Thesen)

16:00 Uhr **Kaffeepause im Foyer Großer Saal**

Chair: Ulrich Stöckle und Frank Marusch
(Ort: Großer Saal, Schloss Neuhardenburg)

16:30 Uhr **Weiterbildungsskooperation aus Sicht eines Grundversorgers**
Stefan Lenz, Kreiskrankenhaus Prignitz
Stefan Farke, St. Marien-Krankenhaus Berlin
(30 min Vortrag + 30 min Diskussion + Abstimmung über Thesen)

17:30 Uhr **Personalrekrutierung und Bindung vor dem Hintergrund des demographischen Wandels (Video)**
Carla Eysel, Charité - Universitätsmedizin Berlin
(30 min Vortrag + 30 min Diskussion + Abstimmung über Thesen)

18:30 Uhr **Resümee Tag 1 + Diskussion**
Johann Pratschke und Ulrich Stöckle

19:00 Uhr **Abendessen in der Großen Orangerie**

2. Tag: Samstag, 04. März 2023

Chair: Sascha Weiß und Eva Dobrindt
(Ort: Großer Saal, Schloss Neuhardenburg)

9:00 Uhr **Talent-Akquisition und Mitarbeiter-Engagement – Denkanstöße**
Stefan Kürbis, Novanta
(30 min Vortrag + 30 min Diskussion + Abstimmung über Thesen)

10:00 Uhr **Frührekrutierungs- und Förderungskonzept für Studierende am CMSC**
Markus Scheibel, Schulthess-Klinik Zürich
(15 min Vortrag + 15 min Diskussion + Abstimmung über Thesen)

10:30 Uhr **Kaffeepause im Foyer Großer Saal**

Chair: Ulrich Adam und Sophie Strozyk
(Ort: Großer Saal, Schloss Neuhardenburg)

11:00 Uhr **Moderne Ausbildungskonzepte**
Moritz Schmelzle, Medizinische Hochschule Hannover
(30 min Vortrag + 30 min Diskussion + Abstimmung über Thesen)

12:00 Uhr **Wo findet noch Weiterbildung statt? – Gründung von Weiterbildungsverbänden**
Johann Pratschke, Charité-Universitätsmedizin Berlin
(15 min Vortrag + 15 min Diskussion + Abstimmung über Thesen)

12:30 Uhr **Resümee Tag 2 + Diskussion**
Sascha Weiß und Ulrich Adam

12:45 Uhr **Verabschiedung und Feedback-Runde**
Johann Pratschke und Ulrich Adam

13:00 Uhr **Lunch**

Anhang 3 – Gesamtheit Statements und Abstimmungsergebnisse

Session I – Flächendeckende Weiterbildung

An welcher Art von Klinik sind/waren Sie aktiv ?
36 Antworten

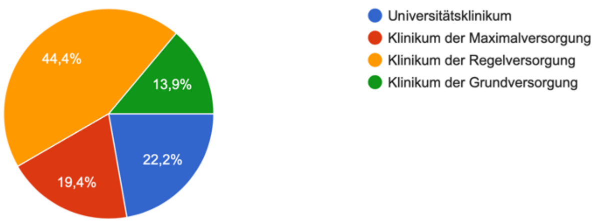

Durch eine Entbürokratisierung des ärztlichen Berufes, kann mehr Zeit für (neue) ärztliche Tätigkeit generiert werden.
36 Antworten

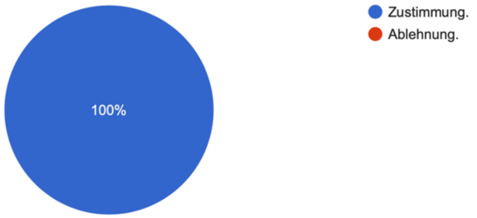

Es sollte ein fester Personalschlüssel (Ärzt:innen zu Patient:innen) für die unterschiedlichen Versorgungslevel definiert werden. Dieser Schüssel soll nicht als Personal-Untergrenze fungieren.
36 Antworten

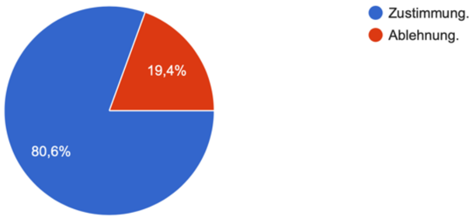

Ärztliche Personalkosten sollten in der Vorhaltepauschale der Gesundheitsreform beinhaltet sein.
36 Antworten

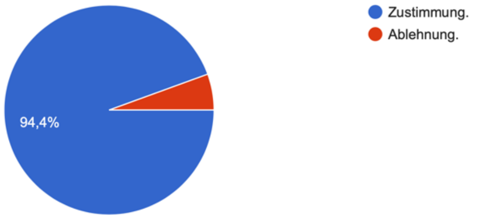

Es sollten vermehrt flexible Arbeitszeitmodelle in der Chirurgie ermöglicht werden und in der Weiterbildungsordnung berücksichtigt werden.

36 Antworten

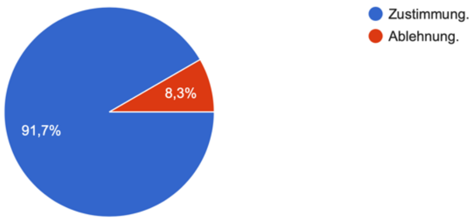

In der chirurgischen Weiterbildung sollte früher eine Wahl zwischen Spezialisierung oder Generalisierung ermöglicht werden.

36 Antworten

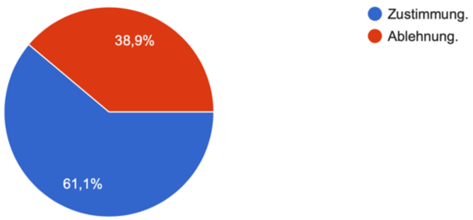

Session II – Weiterbildungskooperationen und Personalrekrutierung

An welcher Art von Klinik sind/waren Sie aktiv ?
37 Antworten

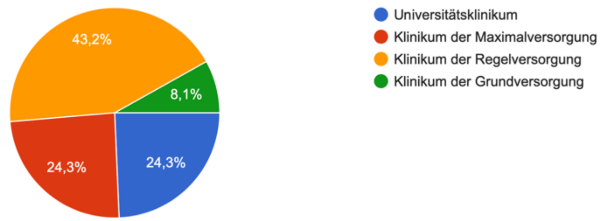

Es sollte eine Integration von definierten externen Rotation in die chirurgische Weiterbildung erfolgen.
37 Antworten

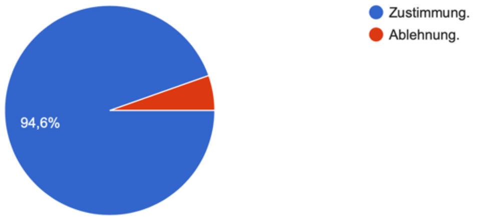

Es sollte eine Integration von Fellowships (Facharztniveau) in die chirurgische Weiterbildung erfolgen.
37 Antworten

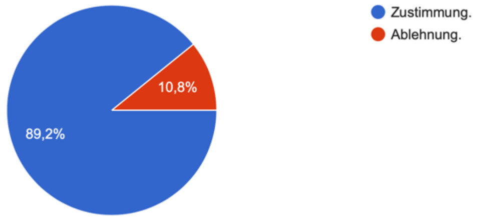

Die Eingriffszahlen in den Weiterbildungskatalogen der chirurgischen Disziplinen sollte reduziert werden.
37 Antworten

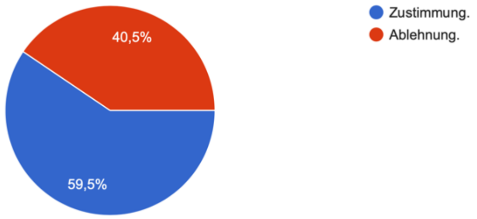

Die Assistenz durch die Weiterbildungsbefugten sollte in der chirurgischen Weiterbildung einen größeren Stellwert einnehmen.
37 Antworten

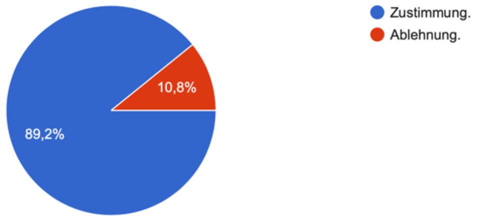

Es sollte strukturierte Integrationsprogamme für chirurgische Weiterbildungsassistent:innen geben.

37 Antworten

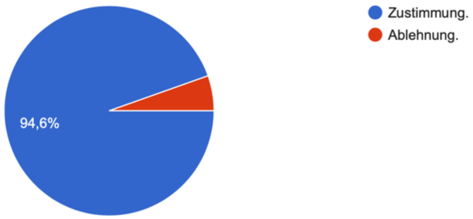

Es sollte differenzierte Weiterbildungsordnungen für für unterschiedliche Versorgungslevels geben.

37 Antworten

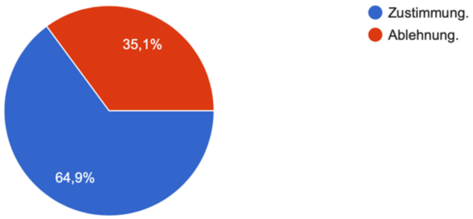

Es sollte kontinuierliches Prozesskostenanalysen und Personalcontrolling geben.

37 Antworten

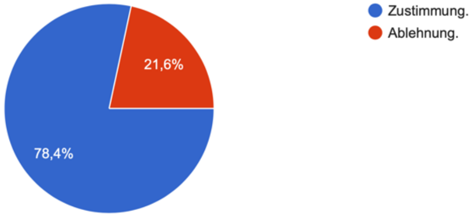

Es soll für Weiterbildungsbefugte ein professionelles Leadership-Training geben.

37 Antworten

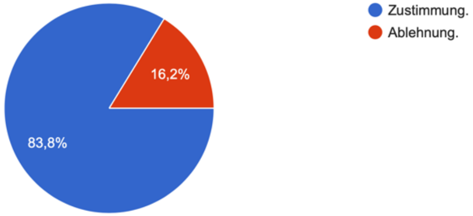

Session III -Talent-Akquistion und Frühantrekrutierung

An welcher Art von Klinik sind/waren Sie aktiv ?

30 Antworten

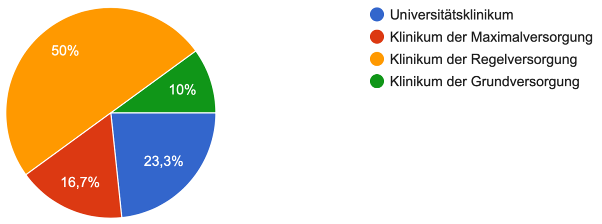

Mitarbeiter-Engagement sollte in der Chirurgie ein größerer Stellenwert eingeräumt werden.

30 Antworten

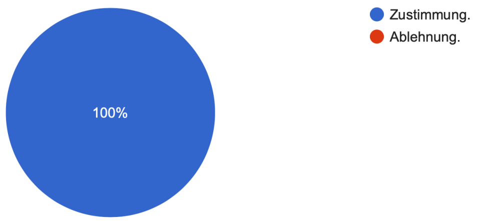

Mitarbeiter-Engagement ist in den chirurgischen Disziplinen umsetzbar.

30 Antworten

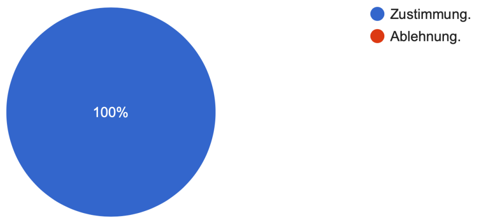

Eine Früh-Rekrutierung von Studierenden für die chirurgischen Disziplinen sollte strukturiert implementiert werden.

30 Antworten

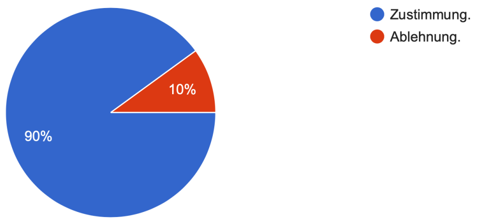

Auch nicht universitäre Chirurg:innen sollte sich in die Früh-Rekrutierung von Studierenden einbringen.

30 Antworten

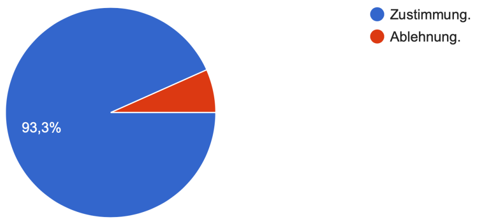

Session IV – moderne Weiterbildungskonzepte und Weiterbildungsverbände

An welcher Art von Klinik sind/waren Sie aktiv ?
26 Antworten

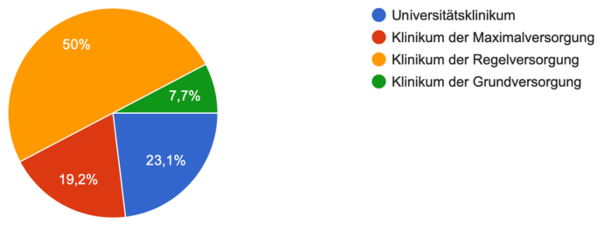

Die Wirtschaftlichkeit steht im Widerspruch zur chirurgischen Weiterbildung.
26 Antworten

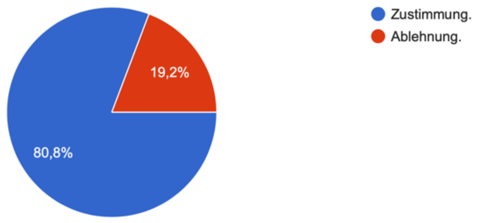

In der Weiterbildung sollte berücksichtigt werden, dass die Qualität der Patientenversorgung oberste Priorität hat, und sich dieser einordnet.
26 Antworten

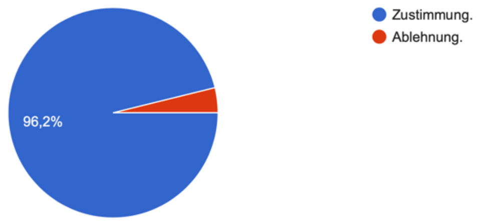

Weiterbildungsverbände sind ein geeignetes Tool um flächendeckend qualitativ hochwertig Weiterbildung anzubieten.
26 Antworten

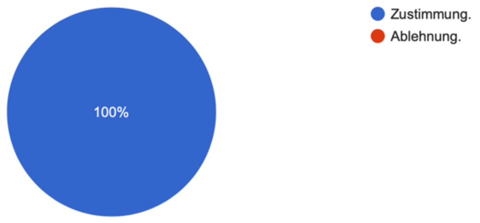

Die BCG wird sich dafür einsetzen diese Weiterbildungsverbände zu implementieren.

26 Antworten

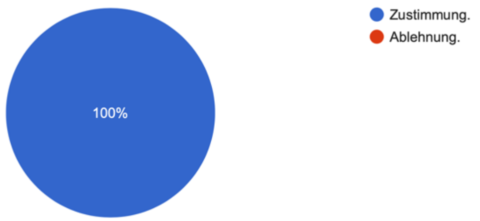

Die BCG soll eine Projektgruppe für die Einrichtung von Weiterbildungsverbänden ins Leben rufen.

26 Antworten

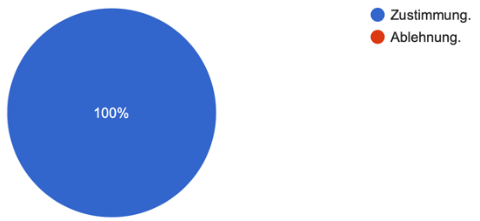

Die BCG sollte sich dafür einsetzen, dass technisch/organisatorische Plattformen in Rahmen von Kooperationsverbänden etabliert werden.

26 Antworten

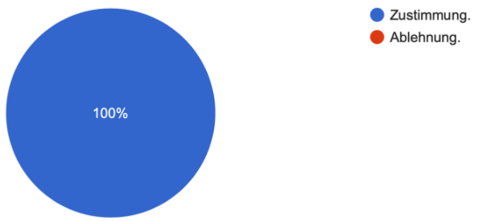

Die Rahmenbedingungen für die Weiterbildungsverbände sollen gemeinsam zwischen der BCG und der Berliner/Brandenburger Ärztekammer definiert werden.

26 Antworten

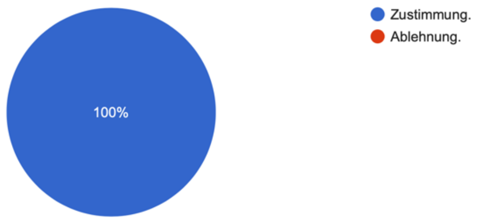

Die Neuhardenberger Gespräche sollten auch in Zukunft genutzt werden um wegweisende Themen der Chirurgie zu diskutieren.
26 Antworten

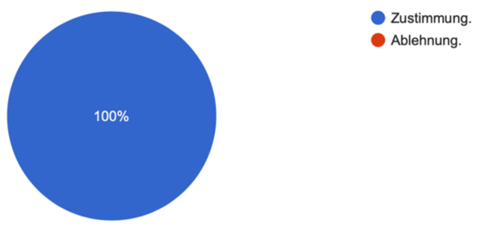

Die Neuhardenberger Gespräche sollten in folgenden Abständen stattfinden.
26 Antworten

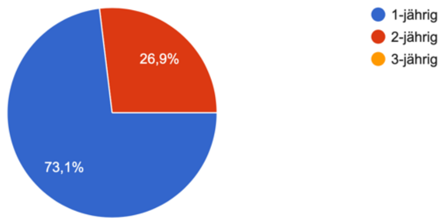

Supplement: Supplementary file 1 — Anhang [file 104_2024_2071_MOESM1_ESM.pdf]
